# Job motivation and associated factors among health workers providing maternal and child health services in Wolaita Zone public hospitals, Southern Ethiopia; A mixed-method study

**DOI:** 10.1371/journal.pone.0320672

**Published:** 2025-05-09

**Authors:** Tamirat Mathewos Milkano, Kassa Daka, Getachew Nigussie Bolado, Mesafint Lukas, Woldetsadik Oshine, Bahilu Balcha

**Affiliations:** 1 Schoolof Public Health, Wolaita Sodo University, Sodo, Ethiopia; 2 School of Nursing, College of Health Science and Medicine, Wolaita Sodo University, Sodo, Ethiopia; 3 BitenaPrimary Hospital, Wolaita Zone health department, Sodo, Ethiopia; 4 Departement of Nursing, School of Nursing and Midwifery, College of Health Science and Medicine, Wachemo University, Hossaina, Ethiopia; Bahir Dar University College of Medical and Health Sciences, ETHIOPIA

## Abstract

**Background:**

The quality of services provided to women, and children is significantly impacted by a lack of motivation and a shortage of competent healthcare staff.Low motivation has a negative impact on the performance of individual healthcare institutions, health workers, patient safety, and the health system as a whole.

**Objectives:**

To assess job motivation and associated factors among health workers providing maternal and child health services in Wolaita Zone public hospitals, Southern Ethiopia, 2023.

**Methodology:**

A facility-based cross-sectional mixed-method study was conducted on randomly selected 319 maternal and child health service providers followed by a purposive sampling technique for the qualitative study. A pretested, structured, self-administered questionnaire obtained from previously conducted studies and in-depth interviews were used to collect quantitative and qualitative studies, respectively. EpiDataV4.6 and Statistical Package for Social Science version 26 were used for quantitative data entry and analysis, respectively, and both bivariable and multivariable logistic regression were done. For qualitative data, OpenCode 4.03 software was utilized to conduct thematic content analysis.

**Result:**

A total of 319 maternal and child health service providers participated in this study, with a 100% response rate. Of them, 142 (44.5%) (95%, CI: 39% - 50%) were motivated. Female gender, payment other than salary not paid on time [AOR (95% CI) 0.159 (0.046–0.549)], work overload[AOR (95% CI) 0.264 (0.083–0.836)], shortage of resources[AOR (95% CI) 0.385 (0.172–0.860)], limited training opportunities[AOR (95% CI) 0.104 (0.030–0.356)], and poor management and leadership of the organizations were statistically significant association between provider’s job motivations.

**Conclusion:**

In this study, nearly forty-five percent of maternal and child health service providers weremotivated. Female gender,payment other than salary not paid on time, work overload, shortage of resources, limited training opportunities, and poor management and leadership of the organizations were significantly associated with providers’ job motivation.Therefore, timely paid other benefitpayments, accessing training opportunities, implementing work force strategy, and availing of resources are very important actions that should be taken.

## Introduction

Motivation is regarded as an essential but complex factor on the effectiveness of health care professionals [1,2], and insufficient motivation has a detrimental effect on an individual health work performances. Globally, the main components of maternity and child health services are doctors, midwives, and nurses, collectively referred to as maternal and child health(MCH) providers) [3]. The quality of services provided to women and children is significantly impacted by a lack of motivation and a shortage of competent healthcare staff [4].

Only 3% of the world’s health workers are located in Sub-Saharan Africa, which has the highest shortage of MCH providers [5,6]. Because of this, maternal mortality in Sub-Saharan Africa is high (542 per 100,000 live births), accounting for 66% (196,000) of all maternal deaths worldwide [7]. When compared to neighboring nations, maternal and child mortality rates in Ethiopia remained high in 2019 [7], at 401 deaths per 100,000 live births and 55 deaths per 1,000 live births, respectively. Unequal allocation of the health workforce and low motivation of health professionals are two frequent issues and difficulties affecting the development of human resources for health [1,8].

Job motivation is also essential for advancing the function of health professionals, strengthening their professional images, improving the healthcare system, raising the standard of treatment, and promoting both personal and community health [9]. The well-known two-factor theory developed by Frederick Herzberg in 1959 postulates that motivation and hygiene are two sets of factors that affect employees’ work attitudes and levels of performance[10].

The delivery of high-quality, efficient, and equitable healthcare is very labor-intensive, and these factors depend on the motivation of healthcare professionals to put out the necessary effort on their jobs [2,11]. According to the World Health Organization (WHO), “a country’s capacity to achieve its health goals depends in great part on the knowledge, skills, motivation, and deployment of those individuals who are in charge of planning and delivering health services”. Another WHO report also states that staff shortages, brain drain, low motivation, and poor performance of human resource issues in sub-Saharan Africa have been so severe that some have called it a “health workforce crisis” in Africa. The motivation of healthcare professionals has been proposed as the primary determinant of the quality of healthcare services, even though there are other causes for this underperformance [12].

Several studies showed that motivated healthcare workers are more likely to apply their knowledge and skills to the actual delivery of healthcare, and they also showed that the successful application of their abilities by a well-motivated health workforce contributes greatly to the provision of high-quality healthcare services [13–16].Health professionals in Ethiopia face major challenges with regard to supply, motivation, retention, distribution, and performance, being hampered by low salaries, limited access to training, and subpar facility infrastructure [17].The various studies carried out in our countries also indicated that low health worker motivation may affect the success of healthcare sector reforms and programs [17,18].

In Ethiopia, several strategies have attempted to address the challenges of job motivation. The strategies include increasing incentives and benefits for healthcare workers by applying the job evaluation and grading system and improving working and living conditions in healthcare facilities by providing supportive supervision. However, these solutions are not sufficient to improve the job motivation of MCH providers [19,20].

There have been a few studies conducted in the country merely on overall healthcare professionals as study participants, which make it difficult to point out the job motivation of maternal and child health care workers. They are limited by focusing on hospital-based workers, who provide the majority of MCH services [17,21–23].However, no study to-date has been conducted on the job motivation and associated factors among health workers providing MCH services in Wolaita Zonepublic health facilities, to the best of our knowledge.Furthermore, most of these previous studies on job motivation of MCH care workers were conducted only by using a quantitative study design, and this is not enough to identify barriers and factors associated with provider’s job motivation. For this reason, this study used a quantitative study augmented with an explanatory sequentialmixed-method approach.Hence, this study aimed to assess job motivation and associated factors among health workers providing MCH services.

The outcome obtained from the present finding improves health service quality and patient satisfaction and contributes to policymakers, researchers, and institutions as a practical guide and baseline data for further development of job motivation among MCH service providers.

## Methods and materials

### Study setting and period

The study was conducted in Wolaita Zone, which is located 396 km south of Addis Ababa and 165km west of Hawassa, South Ethiopia. In Wolaita Zone, there are nine public hospitals namely Wolaita Sodo University Comprehensive and Specialized Hospital, Bodit Primary Hospital, Bale Primary Hospital, Gesuba Primary Hospital, Bombe Primary Hospital, Humbo Primary Hospital, Halale Primary Hospital, Badessa Primary Hospital, and Bitena Primary Hospital.The total numbers of health workers providing maternal and child health service in Wolaita Zone is 1288. The data was taken from a list of the human resource profiles of each hospital. The study was conducted from 1 May to 30 June 2023.

### Study design

A facility-based cross-sectional study design with an explanatory sequential mixed-method approach was conducted in the Wolaita Zonepublic hospitals.

### Source population

All MCH service providers working in public hospitals of Wolaita Zone were source population

### Study population

For a quantitative study, all maternal and child health service providers in Wolaita Zone public hospitals found during the data collection periodand fulfilled the inclusion criteria, and for a qualitative study,head of maternal and child health services in Wolaita Zone public hospitals.

### Eligibility criteria

#### Inclusion criteria.

We have included permanently employed MCH workers with ≥ 6 months of work experience in Wolaita Zone public hospitals in a quantitative study andpermanently employed head providers with ≥6 months of work experience in a qualitative study.

#### Exclusion criteria.

For quantitative study, MCH providers who were practicing for free service in public hospitals were excluded from this study, and for qualitative study, head providerswho had less than 6 months of work experience were excluded.

### Sample size determination and techniques

For the quantitative study, the required sample size was determined by using a single population proportion formula considering the following assumptions: the proportion of overall job motivation in West Arsi Zone was 58.3% [24] with confidence level of 95%, a margin of error (d) of 5%, and a non-response rate of 10%. The total number of health professionals providing maternal and child health services in Wolaita Zone public hospitals was 1288, which was <10,000. Therefore, we used the correction formula, and then, by adding 10% non-response rate, a total sample size of 319 participants.

The sample size for the second objective (associated factors) was determined using the double population proportion formula, and three key factors were taken from previous literature. According to the following assumptions, the sample size was computed using Epi Info version 7.2.4.0 software (stat calc) with the following assumptions: 95% CI, level of required study power of 80%, and ratio of 1:1 (**Table 1**).

**Table 1:** Sample size calculation using Epi info version 7.2.4 software based on associated factors with job motivation of maternal and child health service providers in public Hospitals of Wolaita Zone, 2023.

Variables	Proportion	Power	Ratio	CI (95%)	AOR	Sample Size	References
Workload	26%	80%	1:1	95%	0.461	240	[25]
Facilitytype	84.6%	80%	1:1	95%	0.162	173	[16]
Other benefit	71.9%	80%	1:1	95%	0.281	250	[16]

**Fig 1:**
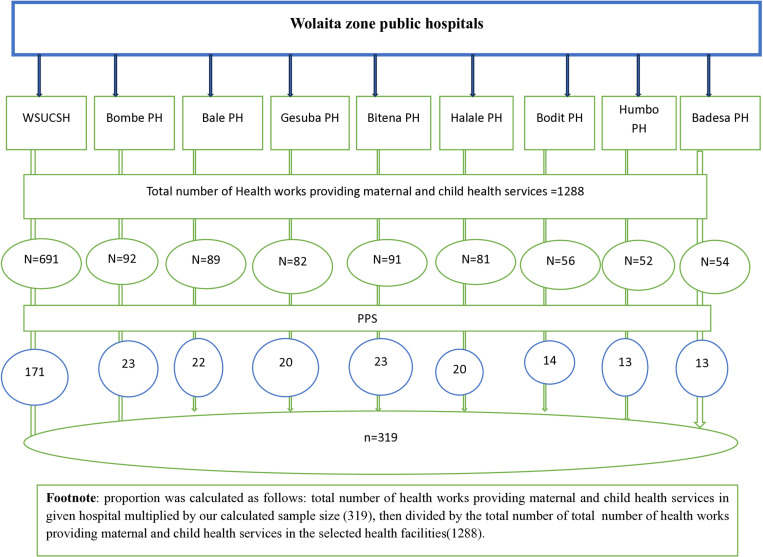
Schematic representation of sampling procedure for job motivation and associated factors among health workers providing maternal and child health services in Wolaita Zone public hospitals, 2023.

Based on the second objective, no sample size was greater than the sample size calculated based on a single population formula. Therefore, the final sample size for this specific study was 319, taken from a single population proportion formula. Then, the determined sample was proportionally allocated to each hospital (i.e., Wolaita Sodo University Comprehensive Specialized Hospital(171), BoditPrimary hospital (14), Bale Primary hospital (22), GesubaPrimary hospital (20), Bombe Primary hospital (23), HumboPrimary hospital (13), HalalePrimary hospital (20), BadessaPrimary hospital (13), and BitenaPrimary hospital (23). Hence, the sample was proportionally allocated to the number of hospitals. Finally, a simple random sampling technique using a lottery method was used to select study units by using their salary payroll lists from the human resource office of each hospital as a sampling frame(**Fig 1).**

For the qualitative study, the purposeful sampling technique was employed for an in-depth interview with selected maternal and child health service heads.A total of 18 head MCH service providers were selected from public hospitals and participated in IDIs until the content reached saturation.

### Study variables

#### Dependent variable.

Job motivation

#### Independent variable.

Socio-demographic related factors: Age, sex, marital status, educational status, professional background, work experience, salary

Individual/personal related factors: other professional respect your profession, opportunities advancement of professionals

Organization related Factors: hospital type, other benefits, salary paid on time, other benefit paid ontime, promotion, workload, necessary resource availability, feedback,remuneration or compensation, career development, reward, training opportunities, work environment, recognition, management, and leadership of the organization.

### Operational definitions

Benefits other than salaries: It is what professionals can get from working institutions other than monthly salary such as overtime payment [26].

Promotion: the advancement of an employee from one job position to another that has a higher income range, a higher-level job title, and, often, more and higher levels of responsibilities [26].

Motivated: If the total summed motivational score is higher than or equal to the 92.38 mean score of the study population [27,28].

Unmotivated: if the total summed score of motivational score is lower than the 92.38 mean score of the study population [27,28].

### Data collection tool and procedures

The data for quantitative study was collected using a structured, self-administered questionnaire. It is adapted with some modifications from a valid and reliable instrument used in previous research done on similar topics by reviewing different literature [17,27,29]. The questionnaire was prepared in the English language, and has four parts: socio-demographic characteristics variables, individual/personal-related variables, organizational-related variables, and motivation status measurement in the form of five-point Likert scales (1 = strongly disagree, 2 = disagree, 3 = neutral, 4 = agree, 5 = strongly agree). This tool was used in different studies conducted in Ethiopia and has a Cronbach’s alpha of 0.888 for internal consistency [17,27]. The data were collected by five BSc nurses, and they had training for two days on how to collect, fill, and handle the data according to the objective of the study. There was continuous monitoring and evaluation by the researchers throughout the data collection.

The data for the qualitative study was collected via in-depth interviews using an interview guide developed from previous studies [17,30]. The interview guide focused on the methods and strategies that each hospital was employing to motivate their maternal and child health care providers. It also explored the overall perception regarding the motivation levels of healthcare professionals, the barriers affecting the motivation of those working in maternal and child health services, and gathered suggestions from interviewees on how the healthcare system could enhance the motivation of its professionals. The in-depth interview was conducted by the authors, and one facilitator was recruited for note-taking. During the interview, each interview was tape-recorded.

### Data processing and analysis

For quantitative data, data-checking processes (sorting and coding) were used to ensure the completeness and consistency of the data. The data was inserted; cleaned, explored for outliers and missed variables, and edited using Epidata version 4.62, and analysis was done using Statistical Package for Social Science (SPSS) version 26 software. A bivariable and multivariable logistic regression analysis was used to see the significant association between the outcome and one or more independent variables. The bivariable logistic regression model’s independent variables that had p-values less than 0.25 were all added to the multivariable logistic regression model. For interpretation, a significant association was identified at an adjusted odds ratio (AOR) with a 95% confidence interval (CI) and a p-value less than 0.05. All the assumptions for binary logistic regression, i.e., model goodness-of-fit and multicollinearity, were checked by using the Hosmer and Lemeshow test, which was 0.47, and the variance inflation factor was 1.94 respectively.Statistical significance was obtained at p-value <0.05. Different frequency tables with the AOR, graphs, charts, and descriptive summaries were used to describe the study variables.

The data analysis for the qualitative study was done using the content-thematic qualitative data analysis method. In-depth interviews were transcribed in the original language, which was Amharic, and translated verbatim to English, by an individual’s expert in both languages. Data analysis was implemented simultaneously with data collection. To become familiar with the data, reading, rereading, and noting down initial ideas were implemented by two independent researchers. Data analysis was done using OpenCode software version 4.03. Then, the data were broken into many sentences and codes were formed, and then content thematic analysis was done for the study. Finally, in order to support or supplement the data from the quantitative analysis, an original verbatim quotation from participants was used.

### Data quality assurance

The data quality for the quantitative study was assured by different methodologies. Data were collected by trained data collectors, and continuous supervision was done on a daily basis. Training was given to equip data collectors about the study objective, informed consent, confidentiality of information, and interview techniques. The tool was pretested on 5% (i.e., 16 providers) of Wachemo University Nigist Eleni Mohamed Memorial Comprehensive Specialized Hospital, which is not found in the study area, to ensure tool validity and to make the researcher familiar with it. From the pretest result, Cronbach’s alpha was computed to assess the internal consistency of the tool (satisfaction items = 0.902, benefits items = 0.932, carefulness items = 0.934, recognition items = 0.783, management/supervision items = 0.831, clear goal items = 0.799, and opportunities items = 0.899), which was acceptable for the population. Ambiguous questions and editorial problems with tools will be modified before actual data collection based on pretest results. The principal investigator and the supervisor were responsible for checking the completeness and quality of the data daily.

To ensure data quality for a qualitative study, two days of intensive reading and understanding of the study objectives, informed consent, confidentiality of information, and interview techniques were done by researchers. Trustworthiness was ensured through prolonged engagement by establishing enough contact with study participants to get an adequate understanding of the concept or to ensure the rigor of the study’s credibility, transferability, dependability, and confirmability were maintained. To ensure credibility, the researcher clarified the method of data analysis, contents of the checklist, and any other issues at the time of the in-depth interview for participants in order to validate the results.

To certify transferability, eighteen experienced head MCH providers recruited from nine public hospitals participated in in-depth interviews (IDIs). Dependability was strengthened by presenting an in-depth description of the processes within the study and other issues that occurred during the interview and throughoutof the study. To warrant confirmability, an investigator had described the purpose of the study, an audio record, the objective of the study, norms for interviews, time allotment, every step of data analysis, and ethically related issues for in-depth interview participants, and the data were preserved.

### Declaration

#### Ethics approval and consent to participate.

Ethical clearance was obtained from the research and ethics committee of the College of Health Sciences and Medicine, Wolaita Sodo University, through an ethical letter with protocol number CRCSD 4/3719/2023. After that, informed written consent was obtained from every study participant prior to data collection, and a letter of cooperation was submitted to the chief executive director of each hospital. Additionally, respondents were told that they might decline the questionnaire and that any information given would be treated with confidentiality to preserve their privacy. Above all, each step of this study was carried out in accordance with the Declaration of Helsinki’s ethical guidelines for medical research on human subjects.

## Results

### Socio demographic characteristics of respondents

A total of 319 maternal and child health providers from the nine Wolaita Zone public hospitals participated in this study, making the response rate 100%. The mean age of the participants was 31.4 ± 6.4SD years and the highest proportions were found within the age range of 20–29 years (47.3%). From the participants, 189 (59.2%) and 190 (59.6%) of the study participants were male and married, respectively, and 245 (76.8%) had a bachelor’s degree. Regarding their professional backgrounds and experiences, 114 (35.7%) and 146 (45.8%) were midwives and had five to ten years of experience, respectively. The majority of participants, 277 (86.8%), had monthly salaries of 6193 ETB and above(**Table 2**).

**Table 2:** Socio-demographic characteristics of maternal and child health service providers in Wolaita Zone public hospital, Southern Ethiopia, 2023.

Variable	Category	Frequency (n)	Percentage (%)
Age	20–29 years	151	47.3
	30–39 years	148	46.4
	40 and above years	20	6.3
Sex	Male	189	59.2
	Female	130	40.8
Marital status	Single	123	38.6
	Married	190	59.6
	Others*	6	1.9
Educational status	Diploma	33	10.3
	Bachelor’s Degree	245	76.8
	Masters and above	41	12.9
Experiences	Below five years	123	38.6
	Five up to ten years	146	45.8
	Above ten years	50	15.6
Professional background	Nurses	99	31
	Midwifery	114	35.7
	Health officers	52	16.3
	General practitioner	45	14.1
	IESO	9	2.8
Salaries	Less than 6193 ETB	42	13.2
	6193 and above ETB	277	86.8

ETB: Ethiopian Birr, IESO: Integrated Emergency Surgical Officers; Others*= divorced and widowed

### Individual/Personal related factors of respondents

The result indicated that 219 (68.7%) of participants got opportunities for advancement as professionals, 253 (79.3%) of participants said other professionals respect their profession, and 66 (20.7%) of participants said other professionals do not respect their profession.

### Organization related factors of respondents

Different organizational factors could influence the motivation of health workers providing maternal and child health services in Wolaita Zone public hospitals. The finding showed that more than one-third, 116 (36.4%), got benefits other than salaries, which is less than 1000 ETB. Of the participants, only 139 (43.6%) responded that their wages were paid on time, and only 40 (12.5%) said their benefits other than salaries, such as duty payments, were paid on time. Of the participants, 258 (80.9%) complained that they hadn’t gotten any feedback from their managers regarding their job within the last six months; however, only 36 (11.3%) were happy with the tasks they performed in their hospitals, while 283 (88.9%) complained about the high workload. Most of the participants, 227 (71.2%), were not satisfied with the present remuneration or compensation, and almost more than one-third of the participants, 238 (74.6%), feel that there is a shortage of the necessary resources needed for their work. Similarly, most of the participants, 269 (84.3%), complained that they were not given any training opportunities, and more than half of the participants, 195 (61.1%), did not enjoy their work environment. As many as 283 (88.7%) respondents were not rewarded for their hard work, but only 36 (11.3%) got a reward for their outstanding performance. Also, 247 (77.4%) of the participants were not promoted for their hard work. Out of the participants, 149 (46.7%) said their management and leadership of the organization were not good(**Table 3**).

**Table 3:** Organization-related factors of health workers providing maternal and child health services in Wolaita Zone public hospital, Southern Ethiopia., 2023.

Variable	Category	Frequency	Percentage
Hospital type	Primary	148	46.4
	Comprehensive and specialized hospital	171	53.6
Benefits other than salaries	Less than 1000 ETB	116	36.4
	1000–2000 ETB	89	27.9
	Greater than 2000 ETB	57	17.9
	Nothing	57	17.9
Salaries paid on time	Yes	139	43.6
	No	180	56.4
Other benefit (duty) paid on time	Yes	40	12.5
	No	279	87.5
Feedback from managerial	Yes	61	19.1
	No	258	80.9
Workload	Happy	36	11.3
	Overload	283	88.7
Remuneration/Compensation	Yes	98	30.7
	No	221	69.3
Necessary resource availability	Yes	81	25.4
	No	238	74.6
Training	Yes	50	15.7
	No	269	84.3
Career development	Yes	228	71.5
	No	91	28.5
Hospital Environment	Favorable	124	38.9
	Not favorable	195	61.1
Promotion	Yes	72	22.6
	No	247	77.4
Reward	Yes	36	11.3
	No	283	88.7
Management and leadership of the organization	Good	31	9.7
	Medium	139	43.6
	Poor	149	46.7

### Job motivation among maternal and child health service providers

**Fig 2:**
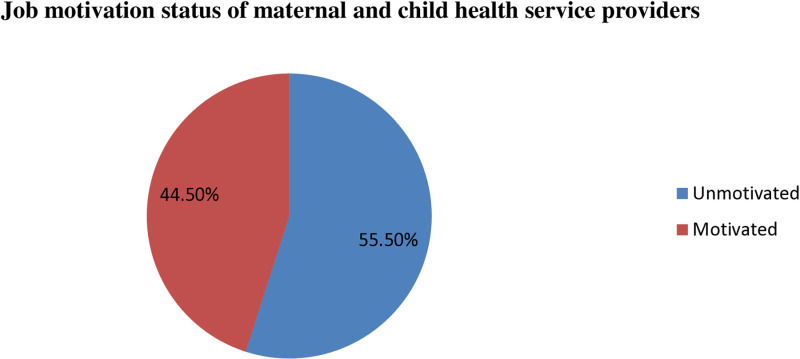
Job motivation among maternal and child health service in Wolaita Zone public Hospitals, Southern Ethiopia, 2023.

The magnitude of job motivation among health workers providing MCH services working in public Hospitals of Wolaita Zone was 44.5% (95%, CI: 39%-50%) (**Fig 2**).

Under satisfaction constructs, four items were loaded. 56 (17.6%) strongly agreed, 85 (26.6%) agreed, 46 (14.4%) were neutral, 29 (29.8%) disagreed, and 37 (11.6%) strongly disagreed with the motivation to work hard.Concerning benefit constructs, 15 (4.7%) of the respondents strongly agreed, 44 (13.8%) agreed, 46 (14.4%) were neutral, 154 (48.3%) disagreed, and 60 (18.8%) strongly disagreed with good employment benefits. Under carefulness constructs, three items were loaded. 91 (28.5%) strongly agreed, 153 (48%) agreed, 39 (12.2%) are neutral, 19 (6%) disagreed, and 17 (5.3%) strongly disagreed with always completing tasks efficiently and correctly.

Concerning management and supervision, 64.9% of the respondents were not satisfied with supervision, and 71.2% said they did not get any regular and timely feedback to help improve performance from their supervisor. (**Table 4**)

**Table 4:** Job motivation among maternal and child health service providers in Wolaita Zone Public Hospital, Southern Ethiopia, 2023.

Constructs	Variables	Strongly disagree		Disagree		Neutral		Agree		Strongly Agree	
		n	%	n	%	n	%	n	%	n	%
Satisfaction items	Motivated to work hard	37	11.6	95	29.8	46	14.4	85	26.6	56	17.6
	Satisfied to work/job	45	14.1	138	43.3	41	12.9	72	22.6	23	7.2
	Work gives personal achievement	30	9.4	111	34.8	57	17.9	89	27.9	32	10.0
	Proud to work in this organization	50	15.7	148	46.4	66	20.7	37	11.6	18	5.6
Benefits	Good employment benefit	60	18.8	154	48.3	46	14.4	44	13.8	15	4.7
	Satisfiedwith payment	129	40.4	131	41.1	33	10.3	18	5.6	8	2.5
	Experience based Payment	100	31.3	108	33.9	55	17.2	49	15.4	7	2.2
	Job to get paid only	65	20.4	128	40.1	60	18.8	51	16.0	15	4.7
Carefulness	I always complete my tasks efficiently and correctly	17	5.3	19	6.0	39	12.2	153	48.0	91	28.5
	Do the thing that needs doing without being asked or told	13	4.1	35	11.0	25	7.8	164	51.4	82	25.7
	I am punctual about coming to work	8	2.5	32	10.0	21	6.6	156	48.9	102	32.0
Recognition	Hardworking of recognized	21	6.6	91	28.5	51	16.0	102	32.0	54	16.9
	Job opportunities for higher-level advancement	41	12.9	114	35.7	50	15.7	78	24.5	36	11.3
Management/ Supervision	Satisfied with supervisor	79	24.8	128	40.1	61	19.1	39	12.2	12	3.8
	Supervisor’s regular and timely feedback	71	22.3	156	48.9	55	17.2	29	9.1	8	2.5
	Good relationswith managers and staff	73	22.9	132	41.4	56	17.6	38	11.9	20	6.3
	Clear organizational mission to staff	44	13.8	110	34.5	77	24.1	75	23.5	13	4.1
	Transparentperformance evaluation	46	14.4	126	39.5	58	18.2	72	22.6	17	5.3
	Clear promotion criteria	29	9.1	131	41.1	66	20.7	76	23.8	17	5.3
	Equal treatment between colleagues	24	7.5	134	42.0	77	24.1	78	24.5	6	1.9
	The Organization provided skill and knowledge	47	14.7	113	35.4	55	17.2	84	26.3	20	6.3
	The institutionus to do our very best on the job	52	16.3	139	43.6	66	20.7	48	15.0	14	4.4
	Work arrangement based on skill, performance,and standard expected from staff	41	12.9	151	47.3	60	18.8	57	17.9	10	3.1
	Cooperation betweenthis organization	35	11.0	152	47.6	66	20.7	57	17.9	9	2.8
	Available tools and materials to use skill full	69	21.6	157	49.2	49	15.4	31	9.7	13	4.1
	Respect and trust by client	30	9.4	45	14.1	60	18.8	143	44.8	41	12.9
Clear goal	Job, duty, requirement, and goal are specific and clear	29	9.1	117	36.7	66	20.7	97	30.4	10	3.1
	Clear objective what to achieve	27	8.5	94	29.5	91	28.5	86	27.0	21	6.6
	There is interference in job	45	14.1	133	41.7	48	15.0	83	26.0	10	3.1
Opportunities	Good opportunity for continuous education	45	14.1	74	23.2	51	16.0	123	38.6	26	8.2
	Adequate in-service training to address the skill gap	63	19.7	160	50.2	58	18.2	27	8.5	11	3.4
	Prefer to continue working in this organization	23	7.2	63	19.7	58	18.2	108	33.9	67	21.0
	As soon I find a better job, I will quite be working this organization	30	9.4	40	12.5	20	6.3	89	27.9	140	43.9

### Factors associated with job motivation

The bivariate logistic regression analysis revealed that sex, marital status, educational status, professional background, professional respect, hospital type, benefits other than salaries, timely salary payment, timely payment of benefits other than salaries, feedback from managers, workload, remuneration and compensation, resource availability, training, career development, hospital environment, promotion, reward, and management and leadership of the organization had a significance level less than 0.25 and were considered candidates for multivariate logistic regression analysis.

In multivariate logistic regression, factors that had a significant association (P-value less than 0.05) with the job motivation of health workers providing maternal and child health services in Wolaita Zone public hospitals were sex, timely payment of benefits other than salaries (overtime payment), work overload, resource availability, training, and management and leadership of the organization.

Those maternal and child health services providers who were female participants were 54.4% less likely to be motivated than male participants, with a p-value of 0.048 [AOR (95% CI) 0.456 (0.209–0.994)]. The study demonstrated that there was also a relationship between timely salary payments and job motivation for maternal and child health service providers. Maternal and child health services providers whose benefits other than salaries paid on time also showed a significant difference in job motivation. Those maternal and child health services providers who had benefits other than salaries not paid on time were 84.1% less likely to be motivated than those who had timely payment, with a p-value of 0.004 [AOR (95% CI) 0.159 (0.046–0.549)]. Those maternal and child health services providers who said workload were 73.6% less likely to be motivated than those who said happy with the tasks they performed, with a p-value of 0.024 [AOR (95% CI) 0.264 (0.083–0.836)].

Similarly, the availability of necessary resources showed a strong relationship between job motivation and maternal and child health service providers. Those maternal and child health services providers who were working in hospitals without sufficient necessary resources were 61.5% less likely to be motivated than those who were working in hospitals with necessary resources, with a p-value of 0.02 [AOR (95% CI) 0.385 (0.172–0.860)]. Training also showed a significant relationship with job motivation among maternal and child health service providers. Those maternal and child health services providers who hadn’t gotten training opportunities were 89.6% less likely to be motivated than those who had gotten training opportunities, with a p-value of <0.001 [AOR (95% CI) 0.104 (0.030–0.356)]. Management and leadership of the organization or hospital also showed a significant association with job motivation. Those maternal and child health services providers who perceived poor management and leadership of the hospital were 80.3% less likely to be motivated than those who perceived good management and leadership of the hospital, with a p-value of 0.039 [AOR (95% CI) 0.197 (0.042–0.918)] (**Table 5).**

**Table 5:** Factors associated with job motivation of bivariable and multivariable logistic regression analysis in Wolaita Zone public hospitals, 2023.

Variable	Category	Motivated	Unmotivated	COR (95% CI)	AOR (95% CI)
Sex	Male	96(30%)	93(29%)	1	1
	Female	46(15%)	84(26%)	0.53 (0.33–0.84)	0.46 (0.21–0.99)
Marital status	Single	45(14%)	78(24.5%)	1	1
	Married	96(30%)	94 (29.5%)	1.77 (1.11–2.82)	1.76 (0.87-3.59
	Divorced	1(0.3%)	5 (1.7%)	0.35 (0.04–3.06)	0.13 (0.04-5.43)
Education status	Diploma	21(6.6%)	12((3.8%)	1	1
	Degree	106(33%)	139(43.7%)	0.44 (0.21–0.93)	0.42 (0.13-1.35)
	Masters &Above	15(4.7%)	26(8.2%)	0.33 (0.13–0.85)	0.39 (0.07-2.33)
Specialty	Nurses	49(15.4%)	50(15.7%)	1	1
	Midwifery	52(16%)	62(19.4%)	0.86 (0.50-1.47)	1.43 (0.62-3.3)
	HO	21(6.6%)	31(9.7%)	0.69 (0.35–1.36)	0.42 (0.14-1.22)
	GP	15(4.7%)	30(9.4%)	0.51 (0.24–1.06)	0.76 (0.22-2.59)
	IESO	5(1.7%)	4(1.4%)	1.28 (0.32–5.03)	2.13 (0.22-20.2)
Respect	Yes	118(37%)	135(42%)	1	1
	No	24(7.5%)	42(13.5%)	0.65 (0.37–1.14)	1.12 (0.48-2.58)
Hospital type	Primary	77(24%)	71(22%)	1	1
	WSUCSH	65(21%)	106(33%)	0.57 (0.36–0.88)	1.72 (0.81-3.64)
Other benefits	< 1000	42(13%)	74(23%)	1	1
	1000–2000 ETB	49(15%)	40(13%)	2.16 (1.23–3.79)	1.34 (0.61-2.95)
	> 2000	36(11%)	21(7%)	3.02 (1.56–5.83)	1.68 (0.65-4.33)
	Nothing	15(5%)	42(13%)	0.63 (0.31–1.27)	0.38 (0.14-1.05)
Salaries paid on time	Yes	87(27%)	52(17%)	1	1
	No	55(17%)	125(39%)	0.26 (0.17–0.42)	0.46 (0.23-1.90)
Other benefit paid on time	Yes	34(10.4%)	6(2%)	1	1
	No	108(34%)	171(53.6%)	0.11 (0.05–0.27)	0.16 (0.05-0.55)
Feedback	Yes	38(12%)	23(7%)	1	1
	No	104(33%	154(48%)	0.41 (0.23–0.73)	1.47 (0.55-3.90)
Workload	Yes	29(9%)	7(2%)	1	1
	No	113(36%)	170(53%)	0.16 (0.07–0.38)	0.26 (0.08-0.84)
Remuneration	Yes	58(18.3%)	40(12.5%)	1	1
	No	84(26.2%)	137(43%)	0.42 (0.26–0.69)	1.09 (0.51-2.34)
Resource availability	Yes	63(20%)	18(6%)	1	1
	No	79(25%)	159(50%)	0.14 (0.08–0.26)	0.39 (0.17-0.86)
Training	Yes	45(14%)	5(2%)	1	1
	No	97(30%)	172(54%)	0.06 (0.02–0.16)	0.11 (0.03-0.36)
Career growth	Yes	114(36%)	114(36%)	1	1
	No	28(8.3%)	63(19.7%)	0.44 (0.27–0.74)	0.83 (0.38-1.80)
Hospital environment	Favorable	78(24.5%)	46(14%)	1	1
	Unfavorable	64(20%)	131(41.5%)	0.29 (0.18–0.46)	0.82 (0.4-1.7)
Promotion	Yes	45(14%)	27(8.5%)	1	1
	No	97(30%)	150(47.5%)	0.39 (0.23–0.67)	1.65 (0.67-4.06)
Reward	Yes	21(6%)	15(5%)	1	1
	No	121(38%)	162(51%)	0.53 (0.26–1.08)	1.37 (0.41-4.59)
Leadership and management	Good	25(8%)	6(2%)	1	1
	Fair	83(26%)	56(17.3%)	0.36 (0.14–0.92)	0.86 (0.2-3.72)
	Bad	34(10.7%)	115(36%)	0.07 (0.03–0.19)	0.19 (0.04-0.92)

***Key:*** *1 = references, COR = Crude Odd Ratio, AOR = Adjusted Odd Ratio, CI = Confidence Interval*

### Qualitative result

**Fig 3:**
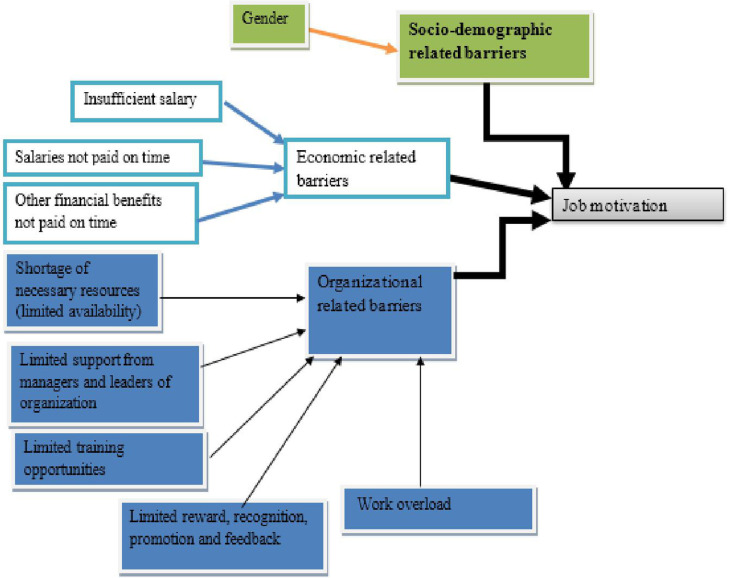
Themes and sub-themes emerged from IDIs for barriers leading to job motivation of health workers providing maternal and child health services in Wolaita Zone public hospital, Southern Ethiopia., 2023.

Eighteen participants, the heads of the maternal and child health services in their respective hospitals, were the participants of IDIs. Of them, 13 had bachelor’s degrees, 3 had master’s degrees, and 2 had diplomas in maternal and child health care. Participants’ ages range from 27 to 36 years old. Using Open Code 4.03 software, the data were categorized into the following three themes based on the information gathered from the IDIs (**Fig 3**).

### Theme one: Socio-demographic related barriers

Many participants in the in-depth interview revealed that being female has an influence on their motivation for the job. They explained that social insecurity and their higher responsibility in caring for their children and their home as a whole may affect their motivation. *“…In our hospital, the female healthcare workforce takes up the lion’s share of total human resources. But when you see it at this level, there is no affirmative action for female professionals. Socially, we may have insecurity. For example, on night duty, there may be threats and dangers posed to us. We have reported this to the concerned bodies, but we have received no better responses from them. It is disappointing for us to be motivated and to focus on our work.” (*a 25-year-old female BSc midwifery professional).

*“…I think we (females) are less motivated than males. This may be because we have more responsibilities at home, such as caring for babies, cooking food for families, cleaning the house, etc. You should focus on your work when you are at work, but we may have divided our attention because we may have a child less than five years old who needs close follow-up and care. This might not motivate female workers more than male workers.”*(a 27-year-old female BSc nurse)*.*

### Theme two: Economic-related barriers

The Majority of the participants explained that their salary level is very low, which is insufficient or not enough to meet their officially defined needs, and even it is not paid on time every month (it may take days to weeks to receive their salary). They also strongly claimed that other financial benefits such as overtime hours or duty payments are not paid on time, and this problem made it difficult to lead their life comfortably.

*“…A professional not being rewarded for his work and duty (overtime) payment not being paid on time can put pressure on his work. As a result, a mother, child, and society as a whole may suffer.*(a 30-year-old male public health officer).

*“…There are many different factors that cause low motivation among maternal and child health service providers. It is said that the patient itself creates a different motivation. Apart from this, there can be many issues that reduce motivation. The leadership, as well as the government, makes job motivation zero. The problem on the part of the government is that it does not pay salaries and overtime payments on time.*(a 29-years-old male medical doctor).

*“*…*I can say that the motivation of the staff in our hospital is low. Also, the job motivation of mothers and children is very low. There is a reason why it is low. For example, not paying overtime, which is part of the regular salary, on time every month plus overtime pay has led to low staff job motivation. We have not been paid any other benefit for a total of 11 months since 2014 E.C. It is one of the things that prevents maternal and child providers from not working if they have good job motivation.*(a 33-year-old male IESO worker).

*“Forprofessionals, other benefit payments are not being paid on time. The work motivation in this institution is getting tired, especially now. In addition, job motivation is very low in our hospital because it will be 2 years if we have not received other benefit payments. For this reason, we have a lot of grievances against all maternal and child providers.* (a32-years old male general practitioner).

### Theme three: Organizational-related barriers

Almost all of the participants in the in-depth interview reported that the hospital has the greatest problem with shortage or scarcity of necessary healthcare resources (limited availability of supplies such as PPE), limited rewards, motivations, promotions, and feedback, limited support from managers and leaders of the organization (the managers and leaders do not work to improve the working environment to make it convenient for healthcare professionals), and limited training opportunities that lead to a lack of updated information, knowledge, and skills for the health workforce.

*“...Since my employment; I have been working with motivation.However, since recently, the hospital has not been able to provide medical supplies for various conditions. So, Iwas not very motivated. It is very difficult to provide maternal and child health services because of inadequate medical equipment, supplies, and utilities like gloves, laboratory reagents, drugs, vital sign material, and electricity. Therefore, lack of laboratory resources, lack of medical equipment, lack of respect for professional benefits, and lack of motivation are the biggest obstacles to providing service.” (*a 30-year-old male doctor)*.*

*“...There are many barriers to maternal and child health care. For example, maternal and child care is one of the government’s focus areas, where it has been decided that resources should be provided free of charge, and we have been working on that. At present, those treatments that we have been providing free of charge have been combined, and before that, subsidies were given to institutions in the form of money. For the past three or two years, due to the denial of those payments, mothers and children have suffered a lot due to the lack of resources.”*(a 31-year-old male BSc midwifery professional).

*“...As an institution, training for all professionals is not provided. It would be good to provide training to implement the policies that come from time to time, provide services, and to give education opportunities for professionals. It could increase motivation but it has not been done.” (*a 27-year-old female midwifery professional*).*

“*…A mother gave birth at home, developed puerperal sepsis, and came to be treated at the private clinic where I work. When I asked her why you gave birth at home, she told me that there are no drugs in the hospital, so they send private clinics to buy drugs. In general, I think the zonal government there did not pay attention to mothers and children.” (*a 30-year-old male doctor*).*

“*…Lack of resources reduces professional motivation because it prevents them from doing their jobs as well as they have learned. In relation to the lack of resources, in this institution there are no resources other than buildings and professionals.The mother who is giving birth comes to health facilities, and the professional loses a glove to deliver her so that the baby’s head is visible, and we will sit and wait until the attendant purchases a glove from a private clinic.” (*a 32-year-old female BSc midwifery professional*).*

“*…We had not given enough training to serve mothers and children. It is good if we have given training to serve both mothers and children because we can add other knowledge to ours, which will help us serve more and increase job motivation.”*(a 29-year-old male BSc nurse professional).

“*…At present, I believe that job motivation has been reduced from time to time due to the fact that we have not received any kind of supportive supervision, feedback, evaluation of performance, or monitoring from management and leadership to create job motivation.”*(a 33-year-old male BSc midwifery professional).

“*…There is a work overload related to the activities of the organization. There is a shortage of health professionals; 24 or more midwives are required, but 5 and 6 midwives are working in this institution. One of the things that prevent me from doing my work systematically is the work overload and shortage of health professionals. The flow of patients is not commensurate with the available professionals. When we see the situation at work, our motivation is low.”* (a 28-year-old female BSc midwifery professional)

## Discussion

This study aimed to assess the job motivation and associated factors of health workers providing maternal and child health services in Wolaita Zone public hospitals. Factors like female gender, timely payment of benefits other than salaries (overtime payment), work overload, resource availability, training, and management and leadership of the organization were significantly associated with the job motivation of MCH providers.

The findings of this study showed that 44.5% of maternal and child health services providers were motivated (95%, CI: 39–50%) towards their job. The findings are in line with the study conducted in Cameroon (42.7%) [31]. However, this finding was significantly higher than that of the study conducted in the Gedeo Zone (19.5%) [32]. This discrepancy might be due to differences in the level of health facilities among the study areas (for instance, the study done in Gedeo Zone comprised 36 public health center professionals, while this study was conducted in one comprehensive and specialized public hospital and eight primary hospitals). Similarly, this incongruence might be due to better quality of health facility infrastructure, attractive incentives, and the presence of regular and ongoing follow-up and intervention to address the problem in the above countries [31].

However, this finding was lower than those of studies conducted in west Amhara (58.6%) [22], Central Ethiopia (63.63%) [17], West Arsi Zone (58.3%) [24], and Jimma Town (55.5%) [27]. The possible reason for this discrepancy might be due to differences in the study area, study period, and study participants (for instance, the study participants for this study were maternal and child health service providers, while the study participants for the study conducted in West Amhara [22] and Jimma Town [27] were all public health professionals and only nurses, respectively). The other plausible reason for this discrepancy might be due to improved health facility infrastructure, working environment, level of health facilities, and technological advancement compared to previous studies.

Sex was the first significant factor associated with maternal and child health service provider job motivation. Those maternal and child health services providers who were female participants were 54.4% less likely to be motivated than those of male participants. This finding is congruent with studies done in Somalia [33] and Gedeo Zone Ethiopia [32], whereas studies in Zambia [29] and Jimma University [34] found that females were more motivated than males. The differences may be due to the weak management system of the health sector in the study area, job insecurity, male higher positions in facilities, a lack of rewards and compensation for outstanding female health workers, and a lack of opportunities for more educational advancement. Therefore, having a strong management system in the health sector, improving job security, providing rewards and compensations, and improving more educational opportunities for female participants might increase their job motivation.This was also supported by the qualitative part of the study, which found that the absence of motivational rewards, compensation, promotion, and job insecurity played a vital role in low motivation among maternal and child healthcare service providers.

The main supporting evidence raised by the participant as social insecurity and their higher responsibility in caring for their children and their home as a whole may affect their motivation. “*In our hospital, the female healthcare workforce takes thehighest role of total human resources. But when you see it at this level, there is no affirmative action for us. Socially, we may have insecurity. For example, on night duty, there may be threats and dangers posed to us. We have reported this to the concerned bodies, but we have received no better responses from them. It is disappointing for us to be motivated and to focus on our work.”*

Timely payment of benefits other than salaries was another significantly associated factor with maternal and child health services provider job motivation. Those maternal and child health services providers whose other benefit payments were not paid on time were 84.1% less likely to be motivated than those whose other benefits were paid on time, with p-values of 0.004 in the [AOR (95% CI) 0.159 (0.046–0.549)]. The findings of this study are similar to those of studies conducted in South Sudan [35] and Jimma town [27]. This might be because maternal and child health service providers become demotivated when their salaries and other incentives are delayed, and they seek alternative sources of funding such as privately-owned businesses, in order to make enough to survive and maintain their standard of living. The government should evaluate and enhance the current payments made to providers of maternity and child health services, standardize salary levels, and act to ensure that medical staff is paid on time. The hospital’s timely payment of salaries and other incentives may increase employees’ motivation for their work. This finding was also supported by the qualitative part of the study. Participants in IDIs raised the concern that delayed payment of benefits other than salary and insufficient salary compared to the high cost of living made them unmotivated towards their job.

Another statistically significant factor in maternal and child health services provider job motivation was work overload. Those maternal and child health services providers who had awork overload were 73.6% less likely to be motivated than those who said they were happy with the tasks they performed. This finding is consistent with studies conducted in some European countries [36], Saudi Arabia [37], Northern Vietnam [38], South Africa [39], and the Gedeo Zone [32]. The reason for this might be a lack of necessary resources for employees, inadequate professional development, and improper implementation of workforce strategies in hospitals. Hiring adequate workers and implementing a work force strategy might improve their job motivation. This finding was supported by the qualitative component, which stated that due to a shortage of staffing, maternal and child health service providers became overwhelmed by a broad range of tasks, such as writing reports, filling forms, attending meetings, and caring for clients with limited time for rest, and this was seen to negatively affect the quality of services and their motivation.

Resource availability was also a statistically significant associated factor with maternal and child health services provider job motivation. Those maternal and child health service providers who said necessary resources were not availed were 61.5% less likely to be motivated than those who had no necessary resources. This is similar to studies done in Nepal [30], West Amhara [22], and Gedeo Zone [32]. This might be due to poor stock management, a lack of budget to purchase, and not prioritizing maternal and child health services. The availability of necessary resources and infrastructure were predictors of motivation [40]. The availability of necessary healthcare resources in the organization might increase their job motivation.

The qualitative part of the study also revealed that the absence of necessary resources, such as personal protective equipment and others was also a major hindering factor in their job. The participants in IDIs reported that it was very difficult to provide maternal and child health services because of inadequate medical equipment, supplies, and utilities like gloves, laboratory reagents, drugs, vital sign material, and electricity. Therefore, lack of laboratory resources, lack of medical equipment, lack of respect for professional benefits, and lack of motivation are the biggest obstacles to providing service.

Similarly, training was a statistically significant associated factor with maternal and child health service provider job motivation. Those maternal and child health services providers who had notraining opportunities were 89.6% less likely to be motivated than those who had training opportunities. This finding is in line with the study conducted in Vietnam [38], Estonia [41], Rwanda [42], Zambia [29], and Gedeo Zone [32]. This might be due to a poor management system, an inappropriate training schedule, and a poor training method. Training opportunities are a crucial human resource management tool to improve or maintain skills, job motivation, work performance, and the quality of health services. The availability of training and educational opportunities for health workers, as well as improving their knowledge and skills, influenced their motivation and work performance [17]. Therefore, having training opportunities for professionals might increase their job motivation. The qualitative part of the study revealed that the hospital has the greatest problem with limited training opportunities that lead to a lack of updated information, knowledge, and skills for the health workforce. It is used to implement policies that come from time to time, to serve both mothers and children more, and to increase job motivation.

The management and leadership of the organization were another statistically significant associated factor with maternal and child health services provider job motivation. Those maternal and child health services providers who perceived poor management and leadership of the organization were 80.3% less likely to be motivated than those who perceived good management and leadership of the organization. This finding is congruent with the study done in some European countries [36], Iran [13], Nigeria [43], West Amhara [22], and Gedeo Zone, Ethiopia [32]. This might be due to a poor management system,not using feedback system, recognition or appreciation, and supervision tools for performance evaluation, and the quality of management and leadership in the organization. So, good management and leadership in the organization might increase their job motivation. This finding is also supported by a qualitative study. Participants in IDIs explained that the management and leadership system in their respective hospitals is unsatisfactory because they haven’t received any kind of supportive supervision, feedback, evaluation of performance, or monitoring from management and leadership to create job motivation.

In last the main theoretical implications of this study which focuses on assessing the job motivation and associated factors of health workers providing maternal and child health services in Wolaita Zone public hospitals, were it is mainly a mixed study and supports the numerical findings by expression of feeling, experience, attitude and emotion and other introvert conditions by IDI toward factors influencing job motivation in public hospitals of Wolaita zone.

Many factors affect maternal and child health services providers’ job motivation based on our study, some of them described here are: the availability of necessary healthcare resources, availability of training opportunities, other benefits paid on time which include per dimes, duty hours, work overload.

To support our study in a theoretical implication these study finding is also associated with variables described by Herzberg’s two-factor theory of motivation. As described by Herzberg’s our motivation and job satisfaction were affected by two types of factors that are Hygiene factor and the motivators. The Hygiene factors are things that people need to have in place which include salary, security, environment, and relationship with coworkers. Whereas motivators also dive workers into motivation and include: recognition, challenging work, personal achievement, responsibilities, and doing meaningful impacts.

This study provides significant theoretical implications for understanding health worker motivation within the framework of **Herzberg’s Two-Factor Theory** and other motivation theories relevant to healthcare settings.By examining the specific factors impacting job motivation among MCH service providers in public hospitals of Wolaita Zone, this research adds to the body of knowledge by offering insights into how **intrinsic** (e.g., job satisfaction, professional respect) and **extrinsic** (e.g., timely payment, resource availability) factors influence motivation. The findings suggest that without addressing these intertwined factors, healthcare workers’ performance and patient care quality may remain suboptimal, highlighting the critical need for organizations to focus on both hygiene and motivational factors in policy formulation.

### Empirical and practical contributions

The study’s empirical findings emphasize the unique structural and resource-based challenges faced by MCH providers, such as resource scarcity and delayed benefits, impacting their motivation. This extends Herzberg’s theory by providing empirical evidence from a healthcare setting in a low-resource environment, suggesting that motivational frameworks must consider contextual factors like resource availability and institutional support, which are especially pertinent in low-income countries.

### Policy and organizational implications

Furthermore, these findings suggest that policies aiming to improve MCH outcomes in resource-constrained environments should prioritize strategies that directly address healthcare workers’ motivation. Such strategies might include institutional support in training and career advancement, timely payment of benefits, and improvement in working conditions. This contributes to the theory of motivation in public health by highlighting the essential role of organizational structures and leadership in enhancing job satisfaction, which is a prerequisite for delivering effective healthcare services.

These theoretical insights underline the complex interplay between individual motivation factors and organizational limitations, advocating for an integrative approach in the health sector to foster sustainable motivation and improve healthcare delivery outcomes.

### Strengthsand limitation of the study

The strengths of this study were that all public hospitals in the Wolaita Zone were included, which makes it more generalizable and representative of the target population, and a mixed-methods approach was used to explore factors that hinder the motivation of maternal and child healthcare providers.

On the contrary, the limitations of this study were that the data for the quantitative part of the study were collected through a self-administered questionnaire, which might be subjected to response bias from the respondents. The other limitation was the cross-sectional nature of the study design; it is difficult to establish cause-and-effect relationships between maternal and child health service providers and job motivation.Next the study lacks generalizability in qualitative concern since it has main differences in sample and study area.

### Conclusion

Nearly forty five percent (44.5%) of the maternal and child health service providers in Wolaita Zone public hospitals weremotivated for their job. Maternal and child health services providers who were female, those whose benefits other than salary was not paid on time, had a work overload, a shortage of necessary resources, a lack of training opportunities, and poor support from the manager and leader of the organization had a statistically significant association with maternal and child health services providers job motivation.

### Recommendation

Hospital management, as well as regional and federal health stakeholders, should pay attention to factors that demotivate maternal and child healthcare service providers, and there should be designed programs to provide affirmative action and improved job security for female professionals, provide in-service training, provide necessary resources such as PPE, and pay salary and other payments such as overtime payments on time, provide regular follow-up and feedback, and provide support and interventions to improve the motivation among service providers. Future researchers are recommended to conduct longitudinal studies to evaluate the cause-and-effect relationship between independent and dependent variables.

## Supporting information

S1 Data SPSS data set of the study. (SAV)

S2 Questionaries Questionaries of the study. (DOCX)

## Abbreviation:

AOR: Adjusted Odds Ratio
CI: Confidence Interval
ETB: Ethiopian Birr
IDIs: In-depth Interviews
MCH: ,Maternal and Child Health
SD: Standard Deviation
SPSS: Statistical Package for Social Science
WHO: World Health Organization
