# Supplementary material for: Job motivation and associated factors among health workers providing maternal and child health services in Wolaita Zone public hospitals, Southern Ethiopia; A mixed-method study
Source: PLoS One. 2025 May 9;20(5):e0320672. doi: 10.1371/journal.pone.0320672 (PMC12063899; doi:10.1371/journal.pone.0320672)
Supplement: S2 Questionaries — (DOCX) [file pone.0320672.s002.docx]

## **Appendixes II: Self-administered Questionnaires**

**Facility Name ________________________________Date__________________**

Instruction: Read each question carefully and circle the option in the column that the best suits your response.

| **Self-administered questionnaires** | | | |
| --- | --- | --- | --- |
| **Part I. Socio demographic data Characteristics** | | | |
| **Code** | **Statements(variables)** | **Response: Circle your choice or write** | **Remark** |
| 101 | Age in year | 1. 20 – 29 |  |
|  |  | 1. 30 – 39 |  |
|  |  | 1. 40 and above |  |
| 102 | Sex | 1. Male |  |
|  |  | 1. Female |  |
| 103 | Marital status | 1. Single |  |
|  |  | 1. Married |  |
|  |  | 1. Divorced |  |
|  |  | 1. Widowed |  |
|  |  | 1. Separated |  |
| 104 | Educational status | 1. Diploma |  |
|  |  | 1. Degree |  |
|  |  | 1. Masters and Above |  |
| 105 | Experience | 1. Below five years |  |
|  |  | 1. five up to ten years |  |
|  |  | 1. Above ten years |  |
| 106 | Professional background | 1. Nurses |  |
|  |  | 1. Midwifery |  |
|  |  | 1. HO |  |
|  |  | 1. General practitioner (GP) |  |
|  |  | 1. IESO |  |
| 107 | Salaries | 1. Less than 6193 |  |
|  |  | 1. 6193 and above |  |

**Part II. Individual/Personal related question**

Read each question carefully and circle the option in the column that the best suits your response.

| **Code** | **Statements(variables)** | **Response: Circle your choice** | **Remark** |
| --- | --- | --- | --- |
| 201 | Opportunities advancement of Professionals | 1. Yes |  |
|  |  | 1. No |  |
| 202 | Professional respect | 1. Yes |  |
|  |  | 1. No |  |

**Part III. Organization related question**

Read each question carefully and circle the option in the column that the best suits your response.

| **Code** | **Statements(variables)** | **Response: Circle your choice** | **Remark** |
| --- | --- | --- | --- |
| 301 | Hospital type | 1. Primary |  |
|  |  | 1. Comprehensive specialized hospital |  |
| 302 | Benefits other than salaries | 1. Less than 1000 ETB |  |
|  |  | 1. 1000 – 2000 ETB |  |
|  |  | 1. Greater than 2000 ETB |  |
|  |  | 1. Nothing |  |
| 303 | Salaries paid on time | 1. Yes |  |
|  |  | 1. No |  |
| 304 | Benefits other than salaries paid on time | 1. Yes |  |
|  |  | 1. No |  |
| 305 | Feedback from managerial last six month | 1. Yes |  |
|  |  | 1. No |  |
| 306 | Work load | 1. Happy |  |
|  |  | 1. Over load |  |
| 307 | Remuneration/Compensation | 1. Yes |  |
|  |  | 1. No |  |
| 308 | Necessary resource availability | 1. Yes |  |
|  |  | 1. No |  |
| 309 | Training | 1. Yes |  |
|  |  | 1. No |  |
| 310 | Career development | 1. Yes |  |
|  |  | 1. No |  |
| 311 | Hospital Environment | 1. Favorable |  |
|  |  | 1. Not favorable |  |
| 312 | Promotion | 1. Yes |  |
|  |  | 1. No |  |
| 313 | Reward | 1. Yes |  |
|  |  | 1. No |  |
| 314 | Management and leadership of organization | 1. Good |  |
|  |  | 1. Fair |  |
|  |  | 1. Bad |  |

**Part IV. Job motivation related question**

Read each question carefully and tick **[√]** against the option that the best suits your response.

| Code | Variables | Strongly agree | Agree | Neutral | Disagree | Strongly disagree |
| --- | --- | --- | --- | --- | --- | --- |
| Satisfaction items | | | | | | |
| 401 | Motivated to work hard | 5 | 4 | 3 | 2 | 1 |
| 402 | Satisfied to work/job | 5 | 4 | 3 | 2 | 1 |
| 403 | Work give personal achievement | 5 | 4 | 3 | 2 | 1 |
| 404 | Proud to work in this organization | 5 | 4 | 3 | 2 | 1 |
| Benefits | | | | | | |
| 405 | Good employment benefit | 5 | 4 | 3 | 2 | 1 |
| 406 | Satisfied with payment | 5 | 4 | 3 | 2 | 1 |
| 407 | Payment is accordance experience | 5 | 4 | 3 | 2 | 1 |
| 408 | Job to get paid only | 5 | 4 | 3 | 2 | 1 |
| Carefulness | | | | | | |
| 409 | I always complete my task efficiently correctly | 5 | 4 | 3 | 2 | 1 |
| 410 | Do thing that needs doing without being asked or told | 5 | 4 | 3 | 2 | 1 |
| 411 | I am punctual about coming to work | 5 | 4 | 3 | 2 | 1 |
| Recognition | | | | | | |
| 412 | Hard working of recognized | 5 | 4 | 3 | 2 | 1 |
| 413 | Job opportunity to higher level advancement | 5 | 4 | 3 | 2 | 1 |
| Management/Supervision | | | | | | |
| 414 | Satisfied with supervisor | 5 | 4 | 3 | 2 | 1 |
| 415 | Supervisor give regular and timely feedback to help improvement of performance | 5 | 4 | 3 | 2 | 1 |
| 416 | Good relation between management and staff | 5 | 4 | 3 | 2 | 1 |
| 417 | Organization mission understood and clear to all staffs | 5 | 4 | 3 | 2 | 1 |
| 418 | Transparency of performance evaluation in organization | 5 | 4 | 3 | 2 | 1 |
| 419 | Health institution promotion criteria clear and understood to all | 5 | 4 | 3 | 2 | 1 |
| 420 | Equal treatment between colleagues | 5 | 4 | 3 | 2 | 1 |
| 421 | Organization provided skill and knowledge | 5 | 4 | 3 | 2 | 1 |
| 422 | The health facility inspires to do very best on job | 5 | 4 | 3 | 2 | 1 |
| 423 | Arrangement on work with skill performance standard expected from staff is clear and understood to all | 5 | 4 | 3 | 2 | 1 |
| 424 | Cooperation between in this organization | 5 | 4 | 3 | 2 | 1 |
| 425 | Available tool and material to use skill full | 5 | 4 | 3 | 2 | 1 |
| 426 | Respect and trust by client | 5 | 4 | 3 | 2 | 1 |
| Clear goal | | | | | | |
| 427 | Job, duty, requirement, and goal are specific and clear | 5 | 4 | 3 | 2 | 1 |
| 428 | Clear objective what to achieve | 5 | 4 | 3 | 2 | 1 |
| 429 | There is interference in job | 5 | 4 | 3 | 2 | 1 |
| Opportunity | | | | | | |
| 430 | Good opportunity to continuous education | 5 | 4 | 3 | 2 | 1 |
| 431 | Adequate in service training to address skill gap | 5 | 4 | 3 | 2 | 1 |
| 432 | Prefer to continue working in this organization | 5 | 4 | 3 | 2 | 1 |
| 433 | As soon I find a better job, I will quite working this organization | 5 | 4 | 3 | 2 | 1 |
